# Academic Stress among High School Students in a Rural Area of Nepal: A Descriptive Cross-sectional Study

**DOI:** 10.31729/jnma.4978

**Published:** 2020-05

**Authors:** Minani Gurung, Natkamol Chansatitporn, Kanittha Chamroonsawasdi, Punyarat Lapvongwatana

**Affiliations:** 1 Faculty of Public Health, Mahidol University, Bangkok, Thailand; 2 Department of Biostatistics, Faculty of Public Health, Mahidol University, Bangkok, Thailand; 3 Department of Family Health, Faculty of Public Health, Mahidol University, Bangkok, Thailand; 4 Department of Public Health Nursing, Faculty of Public Health, Mahidol University, Bangkok, Thailand

**Keywords:** *academic stress*, *adolescents*, *Nepal*

## Abstract

**Introduction:**

The period of adolescence undergoes many physical and mental changes. Changing emotional and physical status along with increasing social, family, and academic pressure lead to various impairments in the mental health of adolescents. Academic failure leads to the suicide rate in adolescents, predominantly high during the declaration of exam results which is significantly high in a rural area in comparison with urban. The study examined the prevalence of academic stress among high school students in a rural area of Rolpa, Nepal.

**Methods:**

A descriptive cross-sectional study was conducted in 6 schools in Rolpa from July to October 2019. The sample size calculated was 521. A convenient sampling technique was used for this study. The target population was adolescents enrolled in high schools of Rolpa. Ethical approval was taken before data collection. The scale for assessing academic stress was used to find out the prevalence. A questionnaire was translated in local language and pre-testing was done in Nepal Police School, Sanga among 10% of the calculated sample size. Data entry was done in Statistical Package for the Social Sciences version 18. Descriptive statistical analysis was done for prevalence calculation. A

**Results:**

Out of a total of 521 students, the prevalence of academic stress was seen among 138 (26.5%) students at a 95% confidence interval (22.72-30.28).

**Conclusions:**

The prevalence of academic stress in our study was high and was consistent with other South Asian studies. Understanding academic stress and providing help and support to the students would help ease the burden for them.

## INTRODUCTION

Academic stress is distress for some anticipated frustration associated with academic failure or even unawareness of the possibility of such failure. A significant source of stress for many school students is academic pressure.^1^ Identified sources of academic-related stress have included fear of falling behind with coursework, finding the motivation to study, time pressures, financial worries, and concern about academic ability.^2^

Academic failure leads to the suicide rate in adolescents, predominantly high during the declaration of exam results which is significantly high in a rural area in comparison with urban.^3^

The main objective of this study is to find out the prevalence of academic stress among high school students in a rural area of Rolpa, Nepal. This study provides baseline information for policymakers to effectively manage and plan academic stress among high school students in Nepal.

## METHODS

A descriptive cross-sectional study was conducted in all high schools of Thawang Rural Municipality, Rolpa, Nepal. Data were collected from six high schools of the municipality from June to August of 2019. Ethical approval was taken from- Committee on Human rights related to Human Experimentation, Faculty of Public Health, Mahidol University (MUPH 2019-054), and Nepal Health Research Council (Reg. no. 233/2019). Approval for data collection was taken from Thawang Rural municipality.

The study populations were grade 11 and 12 students. Each grade had 1-2 classrooms and all the students from grade 11-12 from each classroom participated in the study. Written consent was taken from the parents. The teachers were first contacted and an orientation about the questionnaire, purpose of the study, the importance of privacy, and ensuring the confidentiality of the respondents was done. Secondly, the parents of the students were informed and asked to participate in an orientation program. The parents were then explained about the research and consent was taken from them for their children to be involved in the study. Convenient sampling was done.

Sample size calculation,

n= Z^2^ × p × q / e^2^

= (1.96)^2^ × 0.5 × (1-0.5) / (0.05)^2^

= 384

Where,

n= sample size,
Z= 1.96 for Confidence Interval of 95%,
p= prevalence of academic stress among high school students in Nepal, 0.5,

q= 1-p.

e= margin of error, 5%.

In case of high school students and due to past experiences of drop out cases, incomplete answering, inappropraite answers, answers outside the box that confuse the researcher, scribblind in the options box and many other issues the researcher took a high non-respondent rate of 35% of the sample size. Hence 135 was added.

Therefore, n= 384 + 135= 519. There were total students of 521 in the area and cent percent of data was collected.

Inclusion criteria included students who were aged between 17-19 years of age, enrolled in grades 11-12, willing to participate in the study, and whose parents willingly gave consent. Exclusion criteria included students who were not willing to participate, whose parents did not give consent to be involved in the study and those who did not complete the questionnaire.

The tool used for data collection in the study was a Semi-structured questionnaire with SAAS (Scale For Assessing Academic Stress). SAAS was used to calculate the prevalence of stress among high school students. The semi-structured questionnaire consisted of a socio-demographic profile. The SAAS consisted 30 sets of questions and the total scores were classified into low stress (<22) and high stress (>22) by using the third quartile (75%) as the cutoff point Each level had more sublevis and it was then classified according to various categories based on literature review and standard levels.

The questionnaire was translated to the Nepali language and was sent to review the consistency and contextual meaning of the questions in both languages to the responsible organization. For reliability, data was pretested on 10% of the sample size which was the representative study population other than the sample, to know about the problem related to sequence, meaning, components, and understanding of the questions in a different are than the study area. Another additional editing to the questionnaire was done according to the comments and responses from the pre-test. The questionnaires were self-administered by the students in the class. If any questionnaires were incomplete then it was returned to the student and asked for completion.

Cronbach's Alpha Coefficient test was carried out to ensure the reliability and final adjustment was done accordingly. The alpha coefficient of Cronbach was 0.726 for overall components. The study site was Nepal Police School, Sanga, a site in the outskirt of Kathmandu valley that reflected the population of Rolpa.

Data was entered and exported to a statistical analysis system program (SPSS) version 18. Descriptive analysis was made to assess inconsistencies, outliers, and missing values, and data are presented by tables.

## RESULTS

The overall prevalence of academic stress was 138 (26.5%) at a 95% confidence interval (22.72-30.28). Students answered the SAAS scale and it was seen that many students had low stress which means; they had some form of stress and were not stress-free. The students with high stress were 1 38 (26.5%) out of 521 students (Table 1).

**Table 1:** Prevalence of academic stress.

Variable	n (%)
Low stress	383 (73.5)
High stress	138 (26.5)

The age of the participants ranges from 17 years to 19 years with an average of 18.38 and the standard deviation is .778. Over half the participants are the age of 19 years 294 (56.4%) about one third are from the age of 18 years 131 (25.1%) and the least is from 17 years of age 96 (18.4%) The average age was 18.38±0.778. The high school comprises of grade 11 and 12 in a school. Grade 11 students joined after the board exams and were lower 205 (39%) in number than the grade 12 students 316 (60.7%) Regarding the family type more than half of the participants (62.4%) were joint family which is very common in rural areas of Nepal. Joint families consist of grandparents, uncles, aunts, cousins living under the same roof. In the case of birth order, most of the participants were middle born among their siblings 185 (35.5%). Most of the participants' mothers were either housewives 128 (24.5%) and working in the fields 326 (62.6%). The fathers of the participants were mainly farmers 366 (70.2%). Lastly, regarding family income, there were four categories. The participants with their family income enough with no debt were 297 (57%). The participants with the family income enough with saving were 75 (14.3%) and the ones enough without saving were 74 (14.2%). Similarly, the participants with the family income not enough and with debt were 75 (14.3%). Table 2 shows the general characteristics of the respondents in this study include age, sex, grade, family type, birth order, mother's occupation, father's occupation, and family income (Table 2).

**Table 2:** Socio-demographic profile.

Variables	n (%)
**Age**	
17	96 (18.4)
18	131 (25.1)
19	294 (56.4)
**Sex**	
Male	352 (67.6)
Female	169 (32.4)
**Grade**	
11	205 (39.3)
12	316 (60.7)
**Family type**	
Joint family	325 (62.4)
Nuclear family	196 (37.6)
**Birth order**	
Eldest	176 (33.8)
Middle	185 (35.5)
Youngest	160 (30.7)
**Mother’s occupation**	
Agriculture	326 (62.6)
Housewife	128 (24.5)
Labor	26 (4.9)
Government service	9 (1-7)
Other	4 (0.7)
**Father’s occupation**	
Unemployed	39 (7.5)
Agriculture	366 (70.2)
Labor	54 (10.4)
Government service	47 (9.0)
Other	15 (2.9)
**Family income**	
Enough with saving	75 (14.3)
Enough without saving	74 (14.2)
Not enough but no debt	297 (57)
Not enough with debt	75 (14.3)

## DISCUSSION

The adolescent life stage is a crucial period that makes them highly vulnerable to the various stressors in the environment. The transition is not limited to just the bodily changes and social role, but even at the institutional setting, the transition from high school to higher secondary and even graduate studies require a lot of adjustment and change. High School education in Nepal is a significant turning point in the life of academic students for their further career move. As their educational prerequisites become increasingly tough, students experience stress at this level.

In our study, 26.5% of students had high academic stress. WHO states "the consequences of not addressing adolescents' mental health conditions extend to adulthood, impairing both physical and mental health and limiting opportunities to lead fulfilling lives as adults”.^4^ Academic stress is one of the major factors affecting adolescence. Children these days face more stress due to increased academic curriculum, competition in school, and over-expectation from parents. Our study had academic stress lesser than compared to a study done in 2019 in Banepa, Nepal.^5^

A study conducted in India reported that 66% of students were stressed because of academic pressure, which is significantly higher than our study.^6^

Among the participants, the male-female ratio is almost twice. In the context of rural Nepal, this is a common situation as girls tend to drop out in high school more due to overload of housework, early marriage and pregnancy.^7^

About two-thirds (66%) of the students reported feeling pressure from their parents for better academic performance. A study conducted in Mexico showed about 3% of high stress among high school students which is much lesser than our study. This might be due to different country setting and also the better educational system of Mexico.^8^

Our study has one limitation as some students were discussing the answers even after strictly being told not to do so, the answers were hence answered in agreement and after discussion.

## CONCLUSIONS

The prevalence of academic stress in our study was high and was consistent with other South Asian studies. Our findings are beneficial to educators, parents, and policymakers in Nepal in setting up an effective system to avoid overburdening students. Teachers should be well trained, not only academically but also to detect the signs of stress, burnout, or any problems with students. The school environment should be congenial for adolescents. Students having a problem with academics, catching up with the class, or difficulty in certain classes due to certain teachers or subjects should be paid attention and not neglected. The underlying cause should be addressed with patience. Finally, adolescence is a critical time for formative growth and brain development, hence this period should be well invested. Behaviors that start in adolescence can determine health and well-being for a lifetime.

## Conflicts of Interest:

**None.**
